# Multi-Satellite Relative Navigation Scheme for Microsatellites Using Inter-Satellite Radio Frequency Measurements

**DOI:** 10.3390/s21113725

**Published:** 2021-05-27

**Authors:** Shiming Mo, Xiaojun Jin, Chen Lin, Wei Zhang, Zhaobin Xu, Zhonghe Jin

**Affiliations:** 1 School of Aeronautics and Astronautics, Zhejiang University, Hangzhou 310027, China; moshierming@zju.edu.cn (S.M.); 21924029@zju.edu.cn (C.L.); zwzju@zju.edu.cn (W.Z.); zjuxzb@zju.edu.cn (Z.X.); jinzh@zju.edu.cn (Z.J.); 2 Key Laboratory of Micro-Nano Satellite Research, Hangzhou 310027, China

**Keywords:** multi-satellite relative navigation, radio frequency measurement, distributed multi-satellite range measurement, inter-satellite angle measurement, extended Kalman filter

## Abstract

The inter-satellite relative navigation method—based on radio frequency (RF) range and angle measurements—offers good autonomy and high precision, and has been successfully applied to two-satellite formation missions. However, two main challenges occur when this method is applied to multi-microsatellite formations: (i) the implementation difficulty of the inter-satellite RF angle measurement increases significantly as the number of satellites increases; and (ii) there is no high-precision, scalable RF measurement scheme or corresponding multi-satellite relative navigation algorithm that supports multi-satellite formations. Thus, a novel multi-satellite relative navigation scheme based on inter-satellite RF range and angle measurements is proposed. The measurement layer requires only a small number of chief satellites, and a novel distributed multi-satellite range measurement scheme is adopted to meet the scalability requirement. An inter-satellite relative navigation algorithm for multi-satellite formations is also proposed. This algorithm achieves high-precision relative navigation by fusing the algorithm and measurement layers. Simulation results show that the proposed scheme requires only three chief satellites to perform inter-satellite angle measurements. Moreover, with the typical inter-satellite measurement accuracy and an inter-satellite distance of around 1 km, the proposed scheme achieves a multi-satellite relative navigation accuracy of ~30 cm, which is about the same as the relative navigation accuracy of two-satellite formations. Furthermore, decreasing the number of chief satellites only slightly degrades accuracy, thereby significantly reducing the implementation difficulty of multi-satellite RF angle measurements.

## 1. Introduction

Microsatellites are relatively low cost and have a short development cycle and excellent flexibility. Thus, they are a perfect substitute for traditional large satellites in multi-satellite missions such as satellite formations, especially for large-scale applications. Missions that cannot be achieved using a single satellite can be accomplished with multi-satellite formations through inter-satellite cooperation. Consequently, microsatellite formations are widely employed in a number of space missions.

Inter-satellite relative measurement and navigation are the premise and basis for inter-satellite cooperation in the formation. The traditional method based on ground telemetry, tracking, and command (TT&C) network suffers from limited observation time, low precision, and poor real-time performance, and therefore cannot satisfy the relative navigation application demands for general satellite formations. To meet the high-precision, real-time operations and autonomy requirements of satellite formations, most measurement methods for inter-satellite relative navigation use global navigation satellite systems (GNSS), radar, inter-satellite radio frequency (RF), and optical measurements [1]. Overall, GNSS and RF measurement methods provide the best performance (Table 1) and are widely used. The GNSS-based method has achieved great success, and centimeter-level real-time relative navigation accuracy can be achieved with the carrier phase differential GNSS (CDGNSS) technique for satellite formations in low earth orbit (LEO) [2]. However, the application of the GNSS-based method is restricted because highly accurate measurement cannot be guaranteed in orbits above LEO. This method also offers limited autonomy because the GNSS constellation depends on the ground TT&C network. The inter-satellite RF measurements, which are independent of satellite orbital altitude and are almost independent of any external systems, not only have the potential to achieve higher measurement accuracy than CDGNSS, but also have excellent autonomy. Thus, RF measurements play an important role in the increasing number of satellite formation missions.

Unlike GNSS, which supports three-dimensional navigation, inter-satellite RF measurement is a one-dimensional approach. Thus, RF angle measurements are conventionally combined with RF range measurements to achieve inter-satellite relative navigation.

Two-satellite relative navigation based on a single inter-satellite relative measurement (inter-satellite range measurement, angle measurement, or range-rate measurement) has been extensively studied. For angles-only relative navigation, David C. Woffinden et al. [3] studied the observability criteria; Francisco J. Franquiz et al. [4] proposed optimal range observability maneuvers and trajectory planning methods for spacecraft formations under constrained relative orbital motion; Jianjun Luo et al. [5] developed angles-only relative navigation and guidance coupling algorithm in the context of Clohessy–Wiltshire and Tschauner–Hempel dynamics; and Baichun Gong et al. [6] studied the angles-only relative navigation problem for spacecraft proximity operations when the camera offset from the vehicle center-of-mass allows for range observability. For range-only relative navigation, John A. Christian [7] explored the observability of range-only relative navigation and revealed the multiplicities of possible relative trajectories of various special relative orbits; Yanghe Shen et al. [8] conducted relative orbit determination with quantum ranging, which provides more accurate range measurement than traditional methods; and Daan C. Massen et al. [9] and Frank R. Chavez et al. [10] utilized relative orbital elements instead of Hill coordinates for relative orbit determination. Besides, Cagri Kilic et al. [11] explored the relative navigation of a formation of small satellites using only range-rate measurements that may be acquired using radio hardware already on the spacecraft.

Multi-satellite absolute navigation based on inter-satellite measurements has also been studied by several researchers. Yunpeng Hu et al. [12] proposed a novel solution for autonomous orbit determination for three spacecraft with inertial angles-only measurements, and analyzed the observability. Wei Kang et al. [13] developed the observability theory and estimation algorithms for multi-satellite systems.

However, these navigation algorithms have various limitations, such as strict requirements on the satellite orbit type, which restricts their practical application. For satellite formation missions, NASA developed an RF-based autonomous formation flying (AFF) sensor and applied it to the Space-Technology 3 (ST-3) mission. The AFF sensor can achieve an inter-satellite range measurement accuracy of better than 5 mm and an inter-satellite angle measurement accuracy of better than 1 arcmin (0.017 deg) over a range of 50–1010 m [14]. However, the relative navigation accuracy has not been reported. The PRISMA mission successfully verified the inter-satellite relative navigation based on RF range and angle measurements. The inter-satellite range measurement accuracy was 1 cm, angle measurement accuracy was 0.2 deg, and relative navigation accuracy was approximately 70 cm over a range of 2–4 km [15].

Although relative navigation methods based on inter-satellite RF range and angle measurements have been studied and applied, almost all are for two-satellite formations. Relative navigation methods for multi-satellite formations have barely been studied, which significantly hinders the development of satellite formations.

There are two critical problems in the application of RF-based multi-satellite relative navigation. First, although the current inter-satellite RF angle measurement technology is relatively mature, its implementation is significantly more complex than inter-satellite RF range measurement. For multi-satellite formations, the number of inter-satellite angle measurements soars in power series with the number of satellites. Thus, implementing multi-satellite RF angle measurement becomes difficult when using a microsatellite platform due to the extremely limited computational resources. In a formation consisting of *N* satellites, if inter-satellite angle measurement is required between any two satellites, the number of inter-satellite angle measurements increases dramatically to N(N−1). Wang et al. [16] proposed a multi-satellite relative navigation method based on inter-satellite range and angle measurements. Their approach selects one chief satellite, with the others being deputy satellites, and performs inter-satellite angle measurements between the chief and deputy satellites. This method reduces the difficulty of implementing angle measurements to some extent. However, when the number of satellites is large, it is still challenging to achieve angle measurements between the chief and deputy satellites. Second, there is no multi-satellite RF measurement scheme and corresponding multi-satellite relative navigation algorithm. Although multi-satellite navigation methods were previously studied, they focused on the navigation algorithm layer instead of the measurement layer. Without a feasible multi-satellite RF measurement framework, such navigation algorithms are far from practical application.

To overcome these two problems, a novel multi-satellite relative navigation scheme based on RF measurements is proposed. This scheme uses the concept of dividing a satellite formation into deputy satellites and a small number of chief satellites. The inter-satellite range measurements are performed among all the satellites, whereas the inter-satellite angle measurements are conducted only among the chief satellites. As the number of inter-satellite angle measurements is simply related to the pre-specified number of chief satellites, and is therefore independent of the total number of satellites, the difficulty of achieving inter-satellite angle measurements is significantly reduced and the scalability of the satellite formation is not affected. According to previous research by several of the authors [17], a multi-satellite measurement scheme based on time division multiple access (TDMA) could be adopted to achieve high-precision inter-satellite range measurements while effectively solving the scalability problem of frequency division multiple access (FDMA)-based or code division multiple access (CDMA)-based measurement schemes. Such a scheme would fully satisfy the application requirements of multi-satellite relative navigation described above. Based on this RF measurement scheme and the idea of measuring angles among a small number of chief satellites, a multi-satellite relative navigation algorithm could be designed to construct a high-precision multi-satellite autonomous relative navigation scheme for the large-scale applications of microsatellites.

## 2. Proposed Multi-Satellite Relative Navigation Scheme

The proposed multi-satellite relative navigation scheme employs inter-satellite range and angle measurements. The chief satellites perform both inter-satellite range and angle measurements, while the deputy satellites only perform inter-satellite range measurements. For the range measurements, a distributed multi-satellite measurement scheme proposed by the authors [18] is adopted. Accordingly, *K* time slots are assigned to *K* nodes and the measurement signal is broadcast within each time slot by the corresponding node. At the same time, inter-satellite angle measurements are performed among the chief satellites. The angle and range measurements between two satellites are assumed to be measured simultaneously, which can be achieved with the radio frequency measurement method [15]. The relative state estimation for the chief satellites, accomplished based on the range and angle measurements, provides a spatial position reference for the multi-satellite formation.

**Figure 1:**
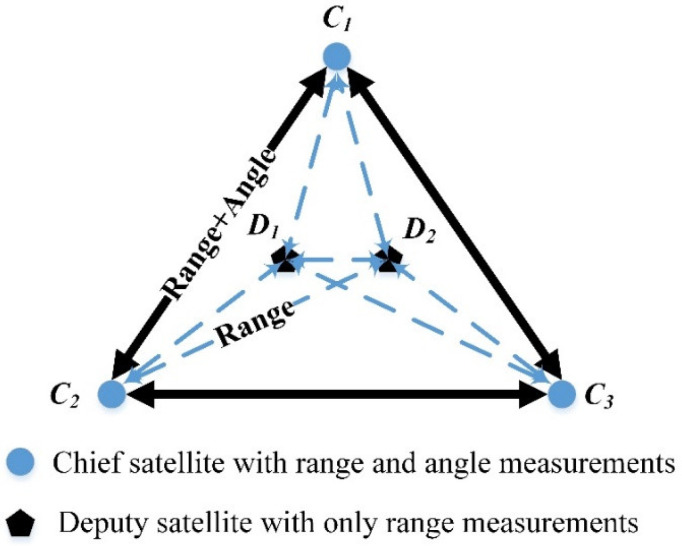
The multi-satellite relative navigation scheme.

Under the assumption that time synchronization, which can be performed with the radio frequency measurement method [18], has been completed among the formation satellites, the relative states of the deputy satellites can be estimated using the trilateral positioning method. Specifically, inter-satellite angle measurements are carried out among the chief satellites ($C_{1}-C_{3}$), and inter-satellite range measurements are conducted among all satellites (Figure 1).

### 2.1. Reference Frame and Relative Orbital Dynamics Modeling

**Figure 2:**
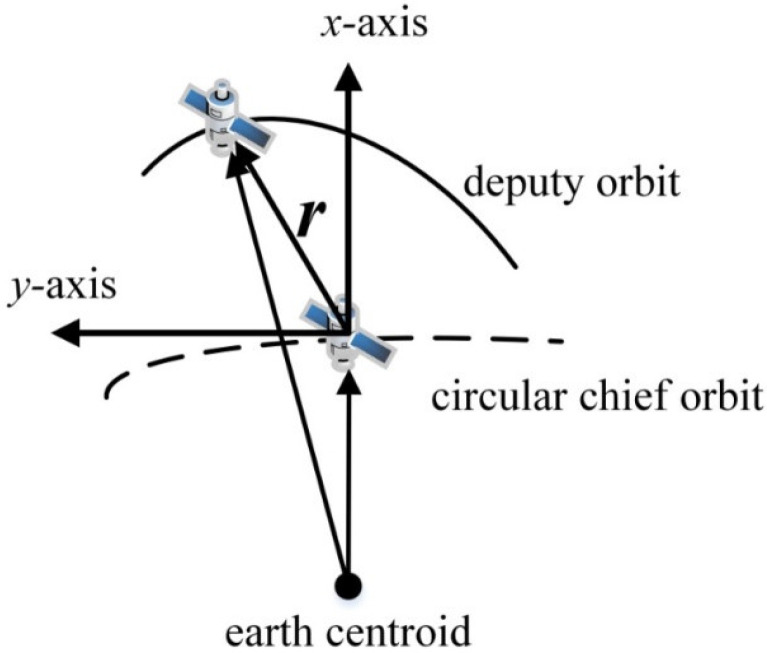
Hill frame is a local vertical, local horizon frame with its origin at the chief satellite. The *z*-axis of the Hill frame is directed out of the page; *r* is the relative position vector.

Clohessy–Wiltshire (CW) equations are widely used to express the linearized spacecraft dynamics in the Hill frame [19]. As shown in Figure 2, the Hill frame is centered on a chief satellite, with the *x*-axis (radius unit vector) aligned with the orbit radius vector, the *z*-axis (normal unit vector) aligned with the orbit angular momentum vector, and the *y*-axis (tangential unit vector) directed so that a right-handed Cartesian reference frame is formed.

The choice of CW dynamics comes with three assumptions: first, the chief satellite’s orbit is approximately circular; second, no disturbing forces are acting on either the chief satellite or the deputy satellites; and third, the spacecraft separation is much smaller than the semi-major axis of the chief satellite’s orbit. The linearized relative orbital dynamics can be expressed with the CW equations in the Hill frame as:

(1) $$\begin{array}{l} \ddot{x}-2n\dot{x}-3n^{2}x=a_{x}\\ \ddot{y}+2n\dot{x}=a_{y}\\ \ddot{z}+n^{2}z=a_{z} \end{array}$$

where ${\left(x,y,z\right)}^{T}$, ${\left(\dot{x},\dot{y},\dot{z}\right)}^{T}$, and ${\left(\ddot{x},\ddot{y},\ddot{z}\right)}^{T}$ denote the relative positions, velocities, and accelerations, respectively. $a_{x},a_{y},a_{z}$ are the perturbing accelerations in the corresponding x-, y- and z-directions. n is the orbital mean motion of the chief spacecraft, which is defined as

(2) $$n=\sqrt{\frac{\mu }{a^{3}}}$$

with μ and a respectively denoting Earth’s gravitational constant and the semi-major axis of the chief satellite’s orbit. To obtain the homogeneous solution of Equation (1), $a_{x},a_{y},a_{z}$ are set to zero, and Equation (1) can be rewritten as

(3) $$\dot{X}(t)=AX(t)$$

where $x={\left(r,v\right)}^{T}={\left(x,y,z,\dot{x},\dot{y},\dot{z}\right)}^{T}$ and

(4) $$A=\left(\begin{array}{cccccc} 0 & 0 & 0 & 1 & 0 & 0\\ 0 & 0 & 0 & 0 & 1 & 0\\ 0 & 0 & 0 & 0 & 0 & 1\\ 3n^{2} & 0 & 0 & 0 & 2n & 0\\ 0 & 0 & 0 & -2n & 0 & 0\\ 0 & 0 & 0 & -n^{2} & 0 & 0 \end{array}\right)$$

The state transition matrix (STM), mapping the state at time $t_{0}=0$ to the state at time *t*, is easily obtained as

(5) $$\Phi (t,0)=e^{At}=(\begin{array}{cccccc} 4-3c_{nt} & 0 & 0 & \frac{s_{nt}}{n} & \frac{2(1-c_{nt})}{n} & 0\\ 6(s_{nt}-nt) & 1 & 0 & \frac{2(c_{nt}-1)}{n} & \frac{4s_{nt}}{n}-3t & 0\\ 0 & 0 & c_{nt} & 0 & 0 & \frac{s_{nt}}{n}\\ 3ns_{nt} & 0 & 0 & c_{nt} & 2s_{nt} & 0\\ 6n(c_{nt}-1) & 0 & 0 & -2s_{nt} & 4c_{nt}-3 & 0\\ 0 & 0 & -ns_{nt} & 0 & 0 & c_{nt} \end{array})$$

where $s_{nt}=sin(nt)$ and $c_{nt}=cos(nt)$.

Considering the limitations of the CW dynamics, some correction methods have been proposed to obtain more precise forms [20,21,22]. These modified CW dynamics can be considered when the CW equations are inappropriate for describing the relative orbital dynamics.

### 2.2. Measurement Modeling

**Figure 3:**
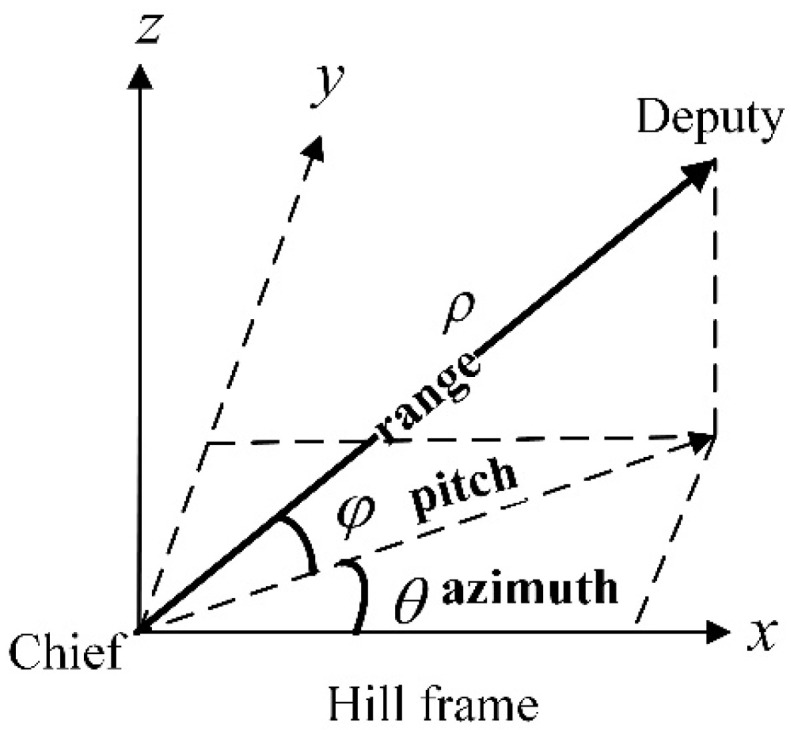
Inter-satellite range and angle measurements in the chief’s Hill frame.

In the proposed multi-satellite relative navigation scheme, inter-satellite angle measurements are performed by the chief satellites and inter-satellite range measurements are performed by both the chief and deputy satellites. The inter-satellite range and angle measurements between the chief and the deputy satellites in the chief’s Hill frame are presented in Figure 3.

#### 2.2.1. Range Measurements

The distributed multi-satellite range measurement scheme is intended to achieve centimeter-level inter-satellite measurements for microsatellite formations, making it suitable for use in a multi-satellite relative navigation algorithm. A brief description of the measurement scheme is as follows.

**Figure 4:**
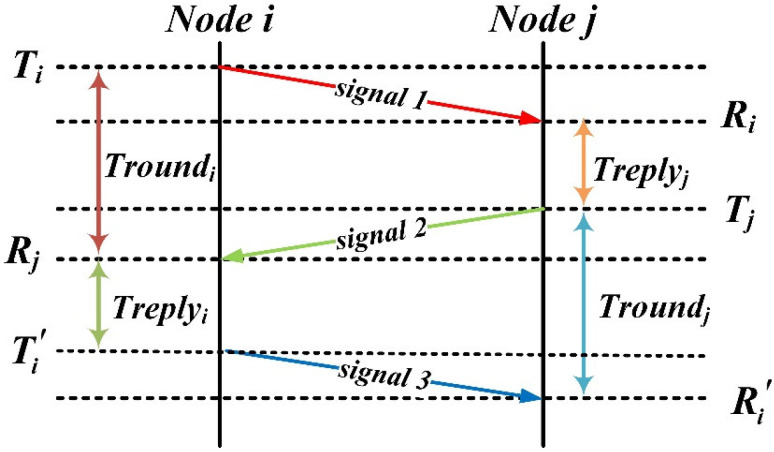
Node-to-node asymmetric double-sided two-way ranging measurement process.

In the distributed multi-satellite measurement scheme, a TDMA-based distributed broadcast protocol is employed in the media access control layer, integrated with the asymmetric double-sided two-way ranging method in the physical layer [17,23]. As shown in Figure 4, the range measurement between nodes *i* and *j* is

(6) $$M_{ij}=\frac{Tround_{i}\times Tround_{j}-Treply_{i}\times Treply_{j}}{Tround_{i}+Tround_{j}+Treply_{i}+Treply_{j}}$$

where $Treply_{i}=T_{i}^{\prime }-R_{j}$; $Tround_{i}=R_{j}-T_{i}$; $Treply_{j}=T_{j}-R_{i}$; $Tround_{j}=R_{i}^{\prime }-T_{j}$; $T_{i}$, $T_{j}$, $T_{i}^{\prime }$ are the signal transmission times; and $R_{i}$, $R_{j}$, $R_{i}^{\prime }$ are the signal reception times.

**Figure 5:**
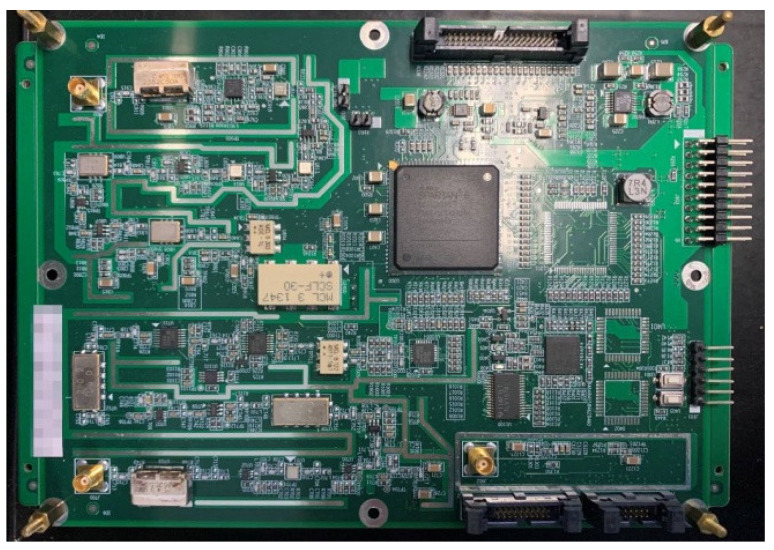
Prototype of RF range measurement equipment for ground testing.

The inter-satellite range measurement equation is

(7) $$f(X)=\rho =\sqrt{x^{2}+y^{2}+z^{2}}$$

where ρ is the inter-satellite range. Our previous numerical simulation results demonstrate that the distributed multi-satellite range measurement scheme achieves high precision and has good scalability [17]. The frequency deviation of the frequency source has a significant effect on the measurement accuracy. Utilizing the miniaturized oven-controlled crystal oscillator (OCXO), commonly used in microsatellite platforms, sub-centimeter-level range measurement accuracy can be achieved. In preliminary ground testing, the measurement accuracy was better than 5 cm (a prototype of the RF range measurement equipment for ground testing is shown in Figure 5), providing a basis for in-orbit application in the future. In Section 4, the range measurement noise parameters are set according to the pre-simulation test results.

#### 2.2.2. Angle Measurements

The bearing angles θ (azimuth angle) and φ (pitch angle) can be used to define the angle measurements, in which the output function is

(8) $$\left\{ \begin{array}{l} \theta =\arctan(\frac{y}{x})\\ \varphi =\arcsin(\frac{z}{\sqrt{x^{2}+y^{2}+z^{2}}}) \end{array}\right.$$

Inter-satellite RF measurement accuracy is related to the carrier frequency. At a distance of 30 km, the inter-satellite angle measurement accuracy can reach 0.1 deg at the L-band frequency and 1 arcmin (0.017 deg) at the Ka-band frequency [14]. Additionally, optional infrared and laser angle measurement methods are available, potentially providing an angle measurement accuracy of 1 arcsec (0.00028 deg) [24,25]. In Section 4, the angle measurement noise parameters are set according to the aforementioned angle measurement accuracy.

## 3. Multi-Satellite Relative Navigation Algorithm

Assuming that a satellite network consists of *N* chief satellites ($C_{i},i=1:N$) and *K* deputy nodes ($D_{j},j=1:K$), the inter-satellite angle and range measurement vector between the chief satellite $C_{i}$ and other chief satellites is expressed as

(9) $$h_{C_{i}}={\left[h_{{}_{C_{i}C_{1}}}^{T},\ldots ,h_{{}_{C_{i}C_{j}}}^{T},\ldots ,h_{{}_{C_{i}C_{N}}}^{T}\right]}^{T},h_{{}_{C_{i}C_{j}}}={\left[\rho_{{}_{C_{i}C_{j}}},\theta_{{}_{C_{i}C_{j}}},\varphi_{{}_{C_{i}C_{j}}}\right]}^{T},i\neq j$$

where $h_{C_{i}C_{j}}$ is the measurement vector from $C_{i}$ to $C_{j}$; $\rho_{C_{i}C_{j}}$,$\;\theta_{C_{i}C_{j}}$, and $\varphi_{C_{i}C_{j}}$ are the range, azimuth angle, and pitch angle, respectively, and are assumed to be measured synchronously. The inter-satellite range measurement vector between deputy satellite $D_{i}$ and the other satellites is

(10) $$\begin{array}{l} h_{D_{i}}={\left[h_{D_{i}C}^{T},h_{D_{i}D}^{T}\right]}^{T}\\ h_{D_{i}C}={\left[\rho_{D_{i}C_{1}},\rho_{D_{i}C_{2}},\ldots ,\rho_{D_{i}C_{N}}\right]}^{T},h_{D_{i}D}={\left[\rho_{D_{i}D_{1}},\ldots ,\rho_{D_{i}D_{j}},\ldots ,\rho_{D_{i}D_{K}}\right]}^{T},i\neq j \end{array}$$

The range measurements are completed using the TDMA-based distributed multi-satellite range measurement scheme, which leads to asynchronization. Thus, epoch naturalization is used to synchronize the range measurements to a reference time. In the proposed relative navigation scheme, the reference time is the end time of the range measurement period.

Any chief satellite can be the origin of the Hill frame. To describe the relative states of the other satellites, $C_{1}$ is adopted as the origin of the Hill frame discussed in Section 2.

At time $t_{k}$, the relative state of satellite S is denoted by $X_{S}^{k}=X_{S}\left(t_{k}\right)$ in the Hill frame; the a priori state estimate and estimated state error covariance matrix are denoted by ${\bar{X}}_{S}^{k}={\bar{X}}_{S}\left(t_{k}\right)$ and ${\bar{P}}_{S}^{k}={\bar{P}}_{S}\left(t_{k}\right)$, respectively, and the a posteriori state estimate and estimation error covariance matrix are denoted by ${\hat{X}}_{S}^{k}={\hat{X}}_{S}\left(t_{k}\right)$ and ${\hat{P}}_{S}^{k}={\hat{P}}_{S}\left(t_{k}\right)$, respectively. The relative state of $C_{1}$ in its own Hill frame is zero, i.e., $X_{C_{1}}={\left(0,\;0,\;0,\;0,\;0\right)}^{T}$.

**Figure 6:**
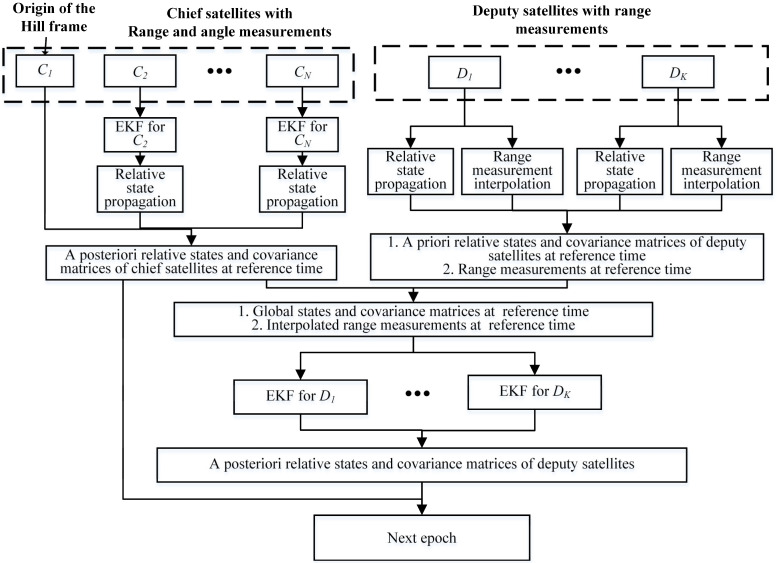
Flowchart of the multi-satellite relative navigation algorithm.

The procedure of the multi-satellite relative navigation algorithm, as shown in Figure 6, involves the following four steps:

- Step 1. Estimate the relative states of the chief satellites based on their range and angle measurements;
- Step 2. Propagate the relative states of the chief and deputy satellites to a reference time;
- Step 3. Obtain the range measurements at the reference time by applying epoch naturalization to the range measurements of all the deputy satellites;
- Step 4. Estimate the relative states of the deputy satellites.

The following pseudo-code describes the process of the multi-satellite relative navigation algorithm in detail (Algorithm 1).
**Algorithm 1.** Multi-satellite relative navigation algorithm.**Input:**  1. The relative state estimates ${\hat{X}}_{C_{i}}^{k-1}$ (*i* = 2:*N*) and ${\hat{X}}_{D_{j}}^{k-1}$ (*j* = 1:*K*) and the corresponding state error covariance matrices ${\hat{P}}_{C_{i}}^{k-1}$ and ${\hat{P}}_{D_{i}}^{k-1}$ at the last epoch $t_{k-1}$;  2. The angle and range measurement vector $h_{C_{1}C_{i}}=\left\{\rho_{C_{1}C_{i}},\varphi_{C_{1}C_{i}},\theta_{C_{1}C_{i}}\right\}$ between the chief satellite $C_{1}$ and $C_{i}$ (*i* = 2:*N*), the corresponding measurement time is $t_{C_{1}C_{i}}$;   3. The range measurement vector of deputy satellite $h_{D_{j}}$ (*j* = 1:*K*), the corresponding measurement time of range measurement between $D_{j}$ and S is $t_{D_{j}S}$;  4. The reference time $t_{ref}$, which is the end time of the current range measurement period;  5. The a priori standard deviation of range and angle measurement noise $\sigma_{\rho }$,$\sigma_{\varphi }$,$\sigma_{\theta }$ and the process noise covariance matrix Q. **Output:**  1. Relative state estimates of all the satellites ${\hat{X}}_{C_{i}}\left(t_{ref}\right)$ (*i* = 2:*N*) and ${\hat{X}}_{D_{j}}\left(t_{ref}\right)$ (*j* = 1:*K*), and the state error covariance matrices ${\hat{P}}_{C_{i}}\left(t_{ref}\right)$ and ${\hat{P}}_{D_{j}}\left(t_{ref}\right)$ at the reference time $t_{ref}$;**Initialize**${\hat{X}}_{C_{i}}^{0}$ (*i* = 2:*N*), ${\hat{X}}_{D_{j}}^{0}$ (*j* = 1:*K*), ${\hat{P}}_{C_{i}}^{0}$, ${\hat{X}}_{D_{j}}^{0}$, $\sigma_{\rho }$, $\sigma_{\varphi }$, $\sigma_{\theta }$, and Q. **for** each chief satellite $C_{i}$ (*i* = 2:*N*) do  Estimate the relative state ${\hat{X}}_{C_{i}}\left(t_{C_{1}C_{i}}\right)$ and the corresponding covariance matrix ${\hat{P}}_{C_{i}}\left(t_{C_{1}C_{i}}\right)$ with the relative navigation algorithm for chief satellite;  Propagate the relative state ${\hat{X}}_{C_{i}}\left(t_{C_{1}C_{i}}\right)$ to the reference time $t_{ref}$ using the CW STM;  Output the relative state ${\hat{X}}_{C_{i}}\left(t_{ref}\right)$ and the corresponding covariance matrix ${\hat{P}}_{C_{i}}\left(t_{ref}\right)$. **end** **for** each deputy satellite $D_{j}$ (*j* = 1:*K*) do  Propagate the relative state ${\hat{X}}_{D_{j}}^{k-1}$ to the reference time $t_{ref}$ using the CW STM **end** **for** each deputy satellite $D_{j}$ (*j* = 1:*K*) do  Interpolate the range measurement vector $h_{D_{j}}$ to reference time $t_{ref}$ with the polynomial interpolation method. The new range measurements are denoted by ${\bar{h}}_{D_{j}}\left(t_{ref}\right)$;  Estimate the relative state of $D_{j}$ based on the interpolated range measurement vector ${\bar{h}}_{D_{j}}\left(t_{ref}\right)$, relative states ${\hat{X}}_{D_{i}}\left(t_{ref}\right)$ (*i* = 1:*N*), and state error covariance matrices ${\hat{P}}_{D_{i}}\left(t_{ref}\right)$ of the chief satellites;  Output the relative state ${\hat{X}}_{C_{i}}\left(t_{ref}\right)$ and state error covariance matrix ${\hat{P}}_{C_{i}}\left(t_{ref}\right)$. **end**

As it uses the distributed multi-satellite range measurement scheme, the multi-satellite relative navigation algorithm is decentralized. That is, each satellite can independently determine its relative state while the other satellites are broadcasting the relative state information.

Regarding the relative navigation algorithm for the chief satellites, both inter-satellite RF range and angle measurements are used. This is the same as the traditional inter-satellite relative navigation algorithm for two-satellite formations. Therefore, the performance of the proposed multi-satellite relative navigation scheme will be demonstrated by comparing the relative navigation simulation results of the deputy satellites with those for the chief satellites.

### 3.1. Relative Navigation Algorithm for Chief Satellites

The inter-satellite measurements between chief satellites $C_{i}$ and $C_{1}$ (the origin of the Hill frame) are:

(11) $$h_{{}_{C_{i}C_{1}}}={\left[\rho_{{}_{C_{i}C_{1}}},\theta_{{}_{C_{i}C_{1}}},\varphi_{{}_{C_{i}C_{1}}}\right]}^{T},i\neq j$$

The Jacobian matrix of the observation equation is

(12) $$H_{{}_{C_{i}}}=\frac{\partial h_{{{}_{C_{i}}}_{C_{1}}}}{\partial X_{{}_{C_{i}}}}{|}_{X_{{}_{C_{i}}}={\bar{X}}_{C_{i}}}$$

The Kalman filter is an optimal linear estimator when the process noise and the measurement noise can be modeled by white Gaussian noise, and the nonlinear problems can be solved with the extended Kalman filter (EKF). Linearization of EKF is carried out through partial derivatives of nonlinear state functions or Taylor series expansion. An alternative to the EKF is the Unscented Kalman Filter (UKF), a recursive estimation filter that meets the requirements of strongly nonlinear systems [26]. While the UKF has drawn more attention and been applied for navigation algorithm [8,13], the EKF, which is widely used in various navigation algorithms, is adopted for the relative navigation of the chief satellites. The state and covariance can be propagated using the analytic solution of the CW STM in the time-update process:

(13) $${\bar{X}}_{{}_{C_{i}}}^{k}=\Phi_{k-1,k}{\hat{X}}_{{}_{C_{i}}}^{k-1}$$

(14) $${\bar{P}}_{{}_{C_{i}}}^{k}=\Phi_{k-1,k}{\hat{P}}_{{}_{C_{i}}}^{k-1}\Phi_{k-1,k}^{T}+Q$$

where $\Phi_{k-1,k}=\Phi \left(t_{k},t_{k-1}\right)$ and Q is the process noise covariance matrix. The nonlinear measurements are used to update the state and covariance in the measurement-update process according to

(15) $$K={\bar{P}}_{C_{i}}^{k}H_{C_{i}}^{T}(W^{-1}+H_{C_{i}}{\left({\bar{P}}_{{}_{C_{i}}}^{k}\right)}^{-1}H_{C_{i}}^{T})$$

(16) $${\hat{X}}_{C_{i}}^{k}={\bar{X}}_{C_{i}}^{k}+K(h_{C_{i}C_{1}}-f({\bar{X}}_{C_{i}}^{k}))$$

(17) $${\hat{P}}_{C_{i}}^{k}=(I-KH_{C_{i}}){\bar{P}}_{C_{i}}^{k}$$

where W denotes the measurement noise, K is the Kalman gain, I is the unit matrix, and f(.) is the output function of the range measurement.

### 3.2. Epoch Naturalization

The distributed multi-satellite range measurement scheme will result in asynchronization of the range measurements among all the satellites. Epoch naturalization is therefore adopted to ensure that the asynchronous range measurements can be used to estimate the relative state of the deputy satellites. For this, polynomial interpolation methods such as Lagrange interpolation, Aitken interpolation, and Newton’s interpolation method are often used [27]. The satellite formation maintenance and control system ensures that the inter-satellite distance is kept within a specific range for a long time. Thus, the range-rate of the inter-satellite distance is very small and remains almost constant over short periods for typical satellite formation-flying configurations (e.g., leader–follower, cartwheel, space-circle) [28]. The Lagrange interpolation method has the advantage of simplicity and is therefore adopted here. The k-degree Lagrange interpolation formula can be expressed as

(18) $$L(x)=\prod_{j=0}^{k}y_{i}l_{j}(x),l_{j}(x)=\prod_{i=0,i\neq j}^{k}\frac{x-x_{i}}{x_{j}-x_{i}},y_{i}=f(x_{i})$$

where x is the independent variable of the interpolation point, $\left(x_{i},y_{i}\right)$ is the *i*-th distinct point for interpolation, and f(x) is a mapping function.

**Figure 7:**
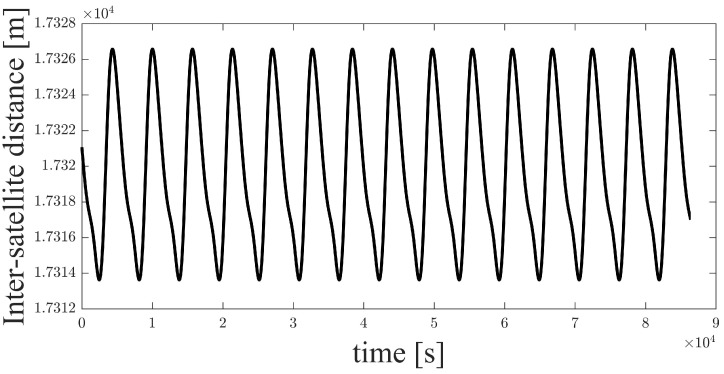
Time history of inter-satellite distance.

**Figure 8:**
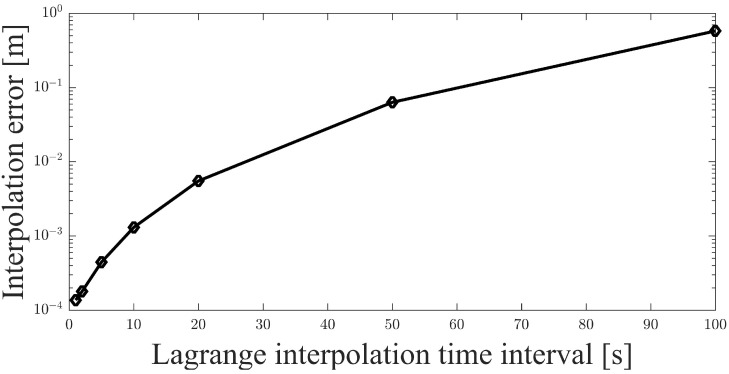
Lagrange interpolation result of inter-satellite distance.

A space-circle formation based on seven-satellite formation is designed for simulations and explained in detail in Section 4. A pre-simulation of interpolation for inter-satellite distance is performed to clarify the impact of interpolation on range measurement accuracy. In the space-circle formation with a space-circle radius of 10 km, the inter-satellite distance between two satellites varies around 17.3 km (Figure 7). The interpolation error is significantly affected by the interpolation interval, which is not greater than the range measurement period. The range measurement period (*T*) varies with the length of the time slot ($t_{slot}$) and the number of satellites in the formation (M), specifically, $T=2Mt_{slot}$. Thus, the distance interpolation simulation is performed to show the effect of the interpolation time interval on the interpolation error. The five-degree Lagrange interpolation is applied in the simulation, and the simulation result is shown in Figure 8.

In Section 4, the time slot in the distributed multi-satellite range measurement scheme is set to 1 s and the number of satellites in the formation is seven. Therefore, the range measurement period is 14 s. As the interpolation interval is not greater than one measurement period, the maximum interpolation time interval is 14 s. When the measurement period is less than 14 s, the interpolation error is within 1 mm (Figure 8), which is negligible in the simulation scenario. Based on the pre-simulation results, five-degree Lagrange interpolation can be adapted to make epoch naturalization in the relative navigation algorithm for deputy satellites.

### 3.3. Relative Navigation Algorithm for Deputy Satellites

The range measurements of each deputy satellite $S_{i}$ are updated after the epoch naturalization and are expressed as

(19) $${\bar{h}}_{D_{i}}={\left[{\bar{\rho }}_{D_{i}C_{1}},{\bar{\rho }}_{D_{i}C_{2}},\ldots ,{\bar{\rho }}_{D_{i}C_{N}},{\bar{\rho }}_{D_{i}D_{1}},\ldots ,{\bar{\rho }}_{D_{i}D_{j}},\ldots ,{\bar{\rho }}_{D_{i}D_{K}}\right]}^{T},j=1:K,j\neq i$$

where $C_{1}$ is the origin of the Hill frame. The Jacobian matrix of the observation equation is

(20) $$H_{D_{i}}=\frac{\partial {\bar{h}}_{D_{i}}}{\partial X_{D_{i}}}{|}_{X_{D_{i}}={\bar{X}}_{D_{i}}}$$

As the aforementioned procedure in Section 3.1, EKF is also adopted to achieve relative navigation for the deputy satellites.

Factors such as angle and range measurement accuracy and the satellites’ spatial configuration affect the relative navigation accuracy of the deputy satellites. Geometric dilution of precision (GDOP) is a term widely used in satellite navigation and geomatics engineering to describe the error propagation of satellites’ geometry on positional measurement precision. It is a critical factor for evaluating the quality of the spatial distribution of the formation satellites and can be obtained from the Jacobian matrix of the observation equation H as

(21) $$GDOP=\sqrt{tr({\left(H^{T}H\right)}^{-1})}$$

where *tr*(∙) denotes the trace of the matrix. GDOP builds a connection between the measurement error and the position error through the relation

(22) $$\sigma_{P}=GDOP\cdot \sigma_{URE}$$

where $\sigma_{P}$ denotes the position estimation error and $\sigma_{URE}$ is the sum of measurement errors [29]. Herein, $\sigma_{URE}$ is the sum of the range measurement error $\sigma_{\rho }$ of the deputy satellite and the position estimation error $\sigma_{e}$ of the chief satellite:

(23) $$\sigma_{URE}^{2}=\sigma_{\rho }^{2}+\sigma_{e}^{2}$$

Due to the lack of precise information, $\sigma_{e}$ can be estimated through the trace of the state covariance matrix as

(24) $$\sigma_{e}^{2}=tr(P_{p}).$$

Therefore, the error covariance matrix of the range measurements can be represented as

(25) $$R_{D_{i}}=diag\{\sigma_{URE_{C_{1}}}^{2},\ldots ,\sigma_{URE_{C_{N}}}^{2},\sigma_{URE_{D_{1}}}^{2},\ldots ,\sigma_{URE_{D_{j}}}^{2},\ldots ,\sigma_{URE_{D_{K}}}^{2}\},j=1:K,i\neq j$$

where $C_{1},\ldots ,C_{N}$ and $D_{1},\ldots ,D_{j},\ldots ,D_{K}$ represent the corresponding satellites.

## 4. Numerical Simulation

**Figure 9:**
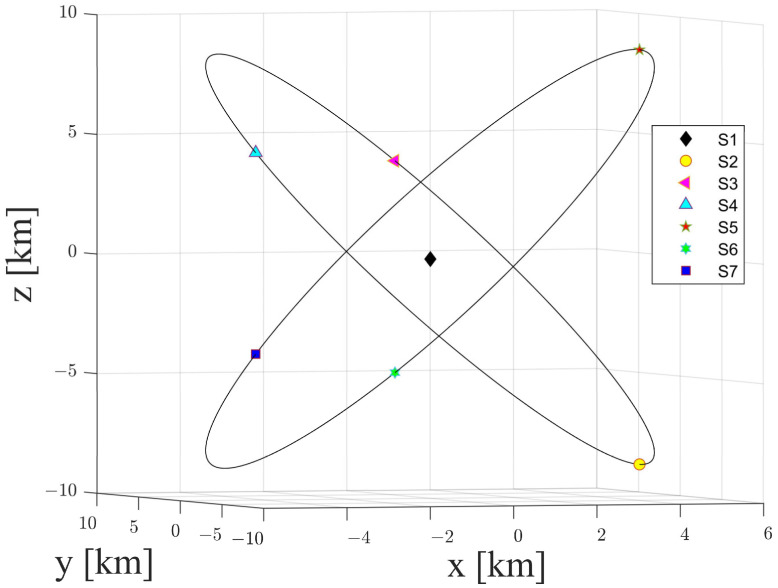
Seven-satellite space-circle formation in the Hill frame centered at $S_{1}$ (space-circle radius of 10 km).

The space-circle formation is a typical satellite formation-flying configuration in which three satellites compose a projected circular formation centered at a reference satellite. To verify the effectiveness of the proposed multi-satellite relative navigation scheme and to evaluate the accuracy of the multi-satellite relative navigation algorithm, we designed a seven-satellite space-circle formation. Precisely, the seven-satellite space-circle formation consists of a reference satellite $S_{1}$ and two space-circle formations (Figure 9). Three of the seven satellites, $S_{2},\;S_{3},\;S_{4}$, are in one relative orbit, with the initial phases being 0 deg, 120 deg, and 240 deg, respectively. The remaining three satellites, $S_{5},\;S_{6},\;S_{7}$, are in the other relative orbit, and their initial phases are 0 deg, 120 deg, and 240 deg, respectively. The orbits of the seven-satellite space-circle formation were calculated for a space-circle radius of 1 km and a radius of 10 km. The results are presented in Table 2 and Table 3.

The number of chief satellites determines the difficulty of achieving inter-satellite angle measurement in a large-scale satellite network. To clarify the influence of the number of chief satellites, simulations were conducted with 2–6 chief satellites:

Case a: 6 chief satellites ($S_{2}$, $S_{3}$, $S_{4}$, $S_{5}$, $S_{6}$, $S_{7}$) and 1 deputy satellite ($S_{1}$);
Case b: 5 chief satellites ($S_{3}$, $S_{4}$, $S_{5}$, $S_{6}$, $S_{7}$) and 2 deputy satellites ($S_{1}$, $S_{2}$);
Case c: 4 chief satellites ($S_{4}$, $S_{5}$, $S_{6}$, $S_{7}$) and 3 deputy satellites ($S_{1}$, $S_{2}$, $S_{3}$);
Case d: 3 chief satellites ($S_{5}$, $S_{6}$, $S_{7}$) and 4 deputy satellites ($S_{1}$, $S_{2}$, $S_{3}$, $S_{4}$);
Case e: 2 chief satellites ($S_{6}$, $S_{7}$) and 5 deputy satellites ($S_{1}$, $S_{2}$, $S_{3}$, $S_{4}$, $S_{5}$).

In Case e, as there are only two chief satellites, no spatial position reference is available, and so the relative navigation algorithm for the deputy satellites diverges according to the theory. Case e is used to validate this inference further.

The measurement accuracy determines the relative navigation accuracy. Pre-simulation analysis and ground tests have shown that sub-centimeter-level range measurement accuracy can be achieved. With the RF measurement method, angle accuracy of 1 arcmin (0.017 deg) to 0.1 deg can be achieved at a working distance of more than 30 km. With the infrared and laser measurement methods, inter-satellite angle measurement accuracy reaches 1 arcsec (0.00028 deg), where the working distance can be up to 30 km for the infrared measurement method and less than 1 km for the laser measurement method.

Inter-satellite RF measurement accuracy is related to factors such as the frequency source, the signal-to-noise ratio, and the inter-satellite distance. To ensure generalization of the simulation results, the range measurement error $\sigma_{\rho }$ is set to

$$\sigma_{\rho }\in \{0.01\;\mathrm{cm},\;0.1\;\mathrm{cm},\;1\;\mathrm{cm},\;10\;\mathrm{cm}\}$$

and the angle measurement errors $\sigma_{\theta }$ and $\sigma_{\varphi }$ are set to

$$\sigma_{\theta }=\sigma_{\varphi }\in \{1\;\mathrm{arcsec},\;0.01\;\deg,\;0.1\;\deg\}$$

Although the inter-satellite angle is measured among all the chief satellites, only the angle measurements between one chief satellite and the other chief satellites are used. Consequently, the redundancy of the angle measurements could be exploited to provide backup information or to improve the navigation accuracy. This is not discussed in the present paper. Without a loss of generality, chief satellite $S_{7}$ is set as the origin of the Hill frame in all the simulations, and the angle measurements between $S_{7}$ and the remaining chief satellites are used.

### 4.1. GDOP Analysis

**Figure 10:**
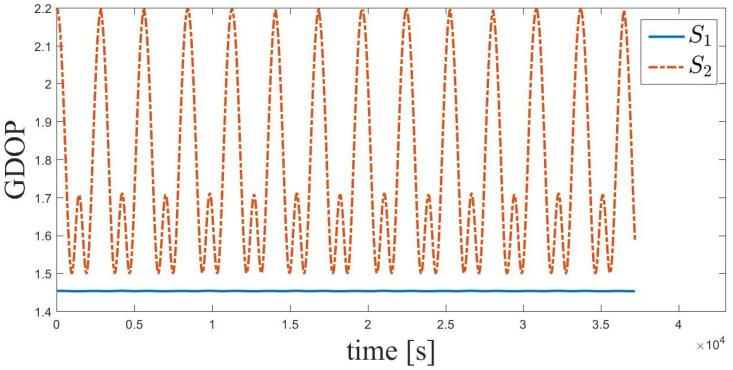
Time history of GDOP of $S_{1}$ and $S_{2}$.

As the structure of the seven-satellite space-circle formation is symmetrical, the geometric locations of satellites $S_{2}\text{–}S_{7}$ can be considered to be equivalent. Take $S_{2}$ as an example. The GDOP values of satellites $S_{2}$ and $S_{1}$ are compared in Figure 10. The mean GDOP value of $S_{1}$ is 1.45, which is significantly smaller than that of $S_{2}$ (mean GDOP value = 1.78). The effect of GDOP on the relative navigation accuracy of deputy satellites is analyzed later.

### 4.2. Simulation of Multi-Satellite Relative Navigation Algorithm

The Satellite Tool Kit (STK) software [30] is used to generate data for the seven-satellite space-circle formation. The initial state error of satellite $S_{i}$ (i=1:6) is set to

$$\Delta X_{S_{i}}^{0}={\left(10,10,10,0.01,0.01,0.01\right)}^{T}$$

The initial state covariance matrix is

$$P_{S_{i}}^{0}=diag\{10^{2},10^{2},10^{2},0.01^{2},0.01^{2},0.01^{2}\}$$

The measurement error covariance matrix of the chief satellite is

$$R_{M}=diag\{\sigma_{\rho }^{2},\sigma_{\theta }^{2},\sigma_{\varphi }^{2}\}$$

and the measurement error covariance matrix of deputy satellite $S_{i}$ is

$$R_{S_{i}}=diag\{\sigma_{URE_{S_{1}}}^{2},\ldots ,\sigma_{URE_{S_{i-1}}}^{2},\sigma_{URE_{S_{i+1}}}^{2},\ldots ,\sigma_{URE_{S_{7}}}^{2}\}$$

The time slot of the distributed multi-satellite measurement scheme is 1 s and the measurement period is 14 s for the seven-satellite formation. The process noise covariance matrix is

$$Q=diag\{0.06^{2},0.06^{2},0.06^{2},0.0024^{2},0.0024^{2},0.0024^{2}\}$$

**Figure 11:**
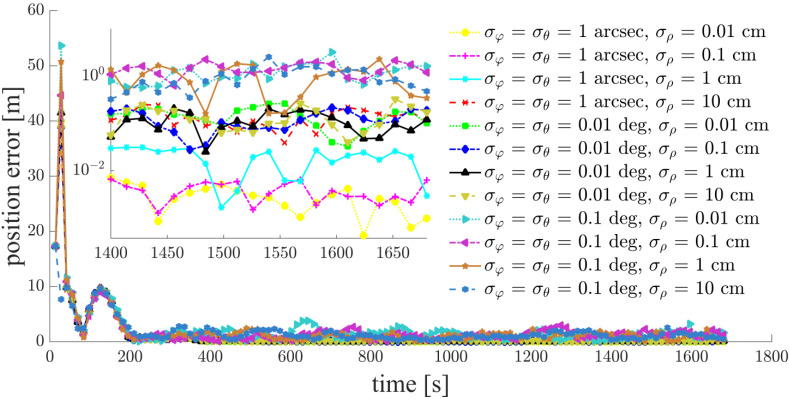
Relative navigation errors of $S_{1}$ (simulated scenario: Case d, space-circle formation radius of 1 km).

**Figure 12:**
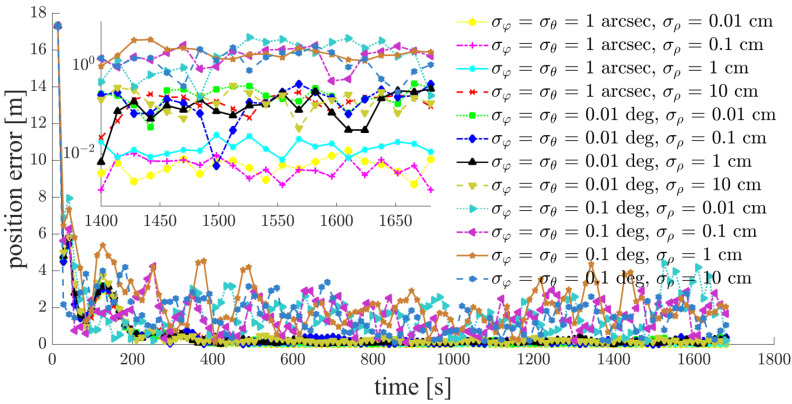
Relative navigation errors of $S_{2}$ (simulated scenario: Case d, space-circle formation radius of 1 km).

**Figure 13:**
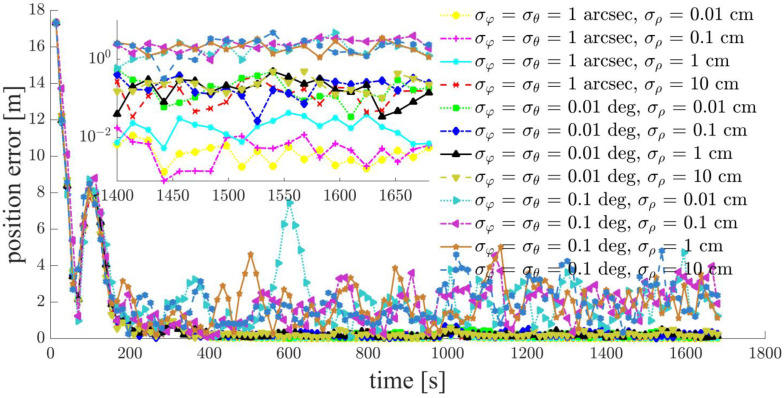
Relative navigation errors of $S_{3}$ (simulated scenario: Case d, space-circle formation radius of 1 km).

**Figure 14:**
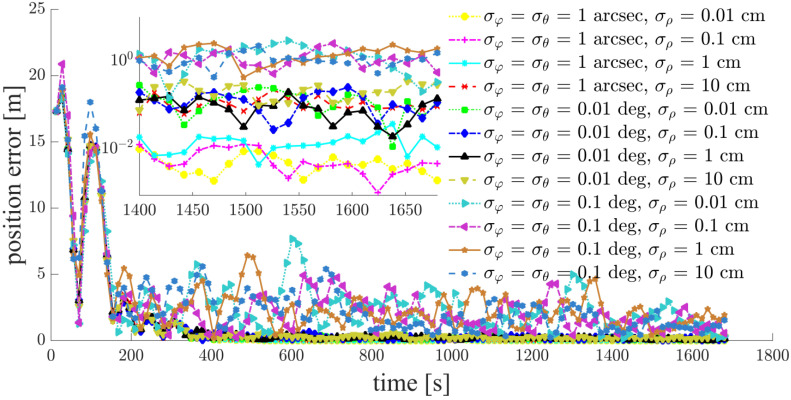
Relative navigation errors of $S_{4}$ (simulated scenario: Case d, space-circle formation radius of 1 km).

**Figure 15:**
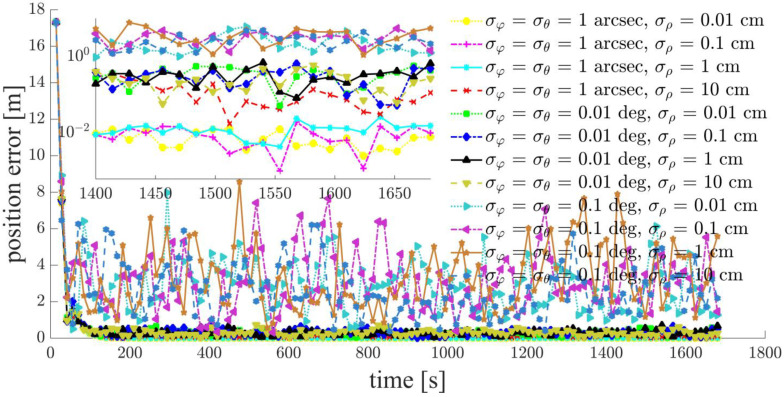
Relative navigation errors of $S_{5}$ (simulated scenario: Case d, space-circle formation radius of 1 km).

**Figure 16:**
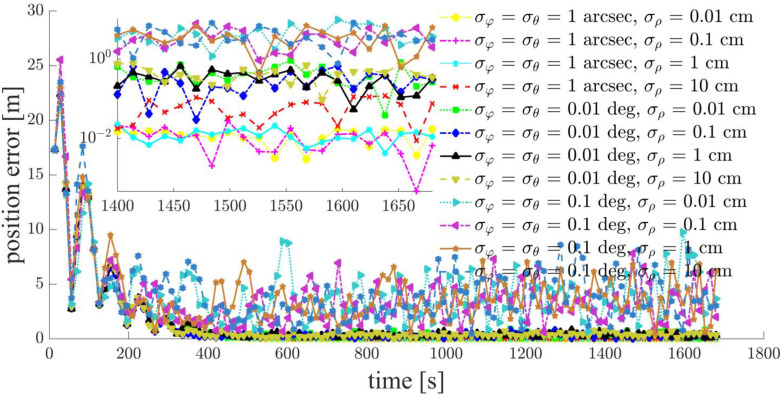
Relative navigation errors of $S_{6}$ (simulated scenario: Case d, space-circle formation radius of 1 km).

Focusing on a typical simulation scenario with a space-circle radius of 1 km and three chief satellites (Case d: $S_{5}$, $S_{6}$, $S_{7}$), the simulation results for the chief satellites $S_{5}$, $S_{6}$ and the deputy satellites $S_{1}$, $S_{2}$, $S_{3}$, $S_{4}$ are shown in Figure 11, Figure 12, Figure 13, Figure 14, Figure 15 and Figure 16. $S_{7}$ is the origin of the Hill frame, and the relative state is $X_{S_{7}}={\left(0,\;0,\;0,\;0,\;0\right)}^{T}$, which does not need to be determined. For brevity, only the statistical results are given for the other cases in the remainder of this paper.

The inter-satellite RF angle measurement accuracy can reach 0.01 deg, and the inter-satellite RF range measurement accuracy is at the centimeter-level for the distributed multi-satellite measurement scheme. Under this condition, the relative navigation results shown in Figure 11, Figure 12, Figure 13, Figure 14, Figure 15 and Figure 16 are summarized in Table 4.

**Figure 17:**
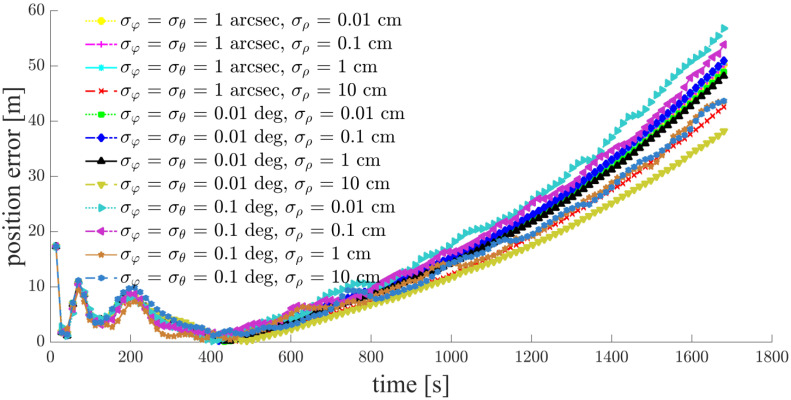
Relative navigation errors of $S_{1}$ (simulated scenario: Case e, space-circle formation radius 1 km).

As inferred above, the relative navigation algorithm for the deputy satellites will diverge in Case e; this is verified by the simulation results in Figure 17. Therefore, no statistics are presented for Case e.

**Figure 18:**
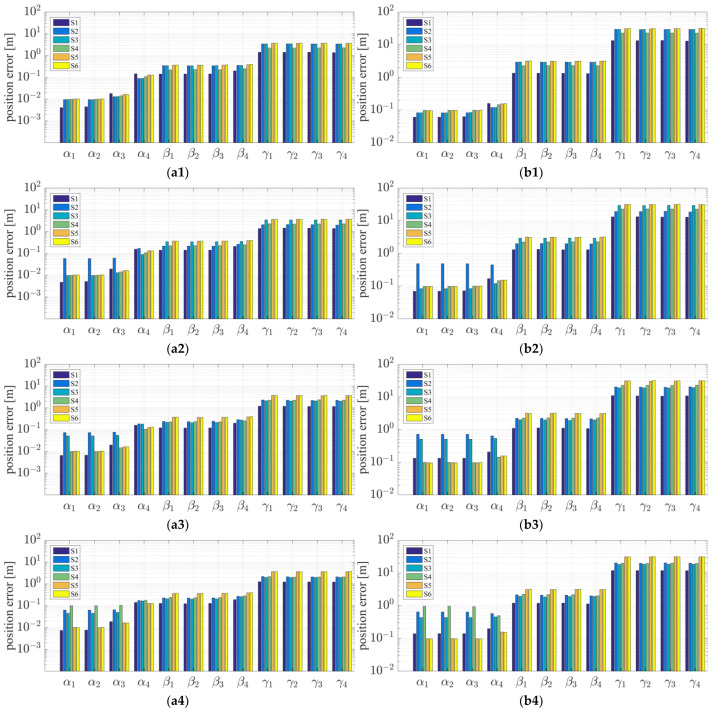
Statistics on the relative navigation accuracy under different simulation scenarios: (**a1**) Case a: space-circle radius 1 km and 6 chief satellites; (**b1**) Case a: space-circle radius 10 km and 6 chief satellites; (**a2**) Case b: space-circle radius 1 km and 5 chief satellites; (**b2**) Case b: space-circle radius 10 km and 5 chief satellites; (**a3**) Case c: space-circle radius 1 km and 4 chief satellites; (**b3**) Case c: space-circle radius 10 km and 4 chief satellites; (**a4**) Case d: space-circle radius 1 km and 3 chief satellites; (**b4**) Case d: space-circle radius 10 km and 3 chief satellites.

To assess the effect of the inter-satellite measurement accuracy, inter-satellite distance, GDOP value, and number of chief satellites on the relative navigation accuracy, simulations were also conducted under the scenarios summarized in Table 5. Statistical results for the relative navigation accuracy are displayed in Figure 18.

Based on the simulation results, we present the following conclusions and analyses:

- The relative navigation algorithm for the deputy satellites converges when there are more than three chief satellites (Figure 11); otherwise, the algorithm diverges (Figure 17).
- Inter-satellite distance is an essential factor in determining the accuracy of multi-satellite relative navigation (Figure 18). The multi-satellite relative navigation accuracy is negatively related to the inter-satellite distance.
- According to the relative navigation simulation results of deputy satellites $S_{1}$ and $S_{2}$ (Figure 18), with a smaller GDOP value, the relative navigation accuracy of $S_{1}$ is remarkably better than that of $S_{2}$.
- Under the typical scenario of $\sigma_{\varphi }=\sigma_{\theta }=0.01\;\deg,\sigma_{\rho }=1\;\mathrm{cm}$ and a space-circle radius of 1 km, the relative navigation accuracy of the deputy satellites is better than that of the chief satellites when there are at least three chief satellites (Table 4 and Figure 18). This is because the relatively low angle measurement accuracy affects the relative navigation accuracy of the chief satellites rather than that of the deputy satellites. When the angle measurement accuracy reaches 1 arcsec and the range measurement accuracy is better than 1 cm, the relative navigation accuracy of all deputy satellites except $S_{1}$ is slightly lower than that of the chief satellites. However, the relative navigation accuracy of the deputy satellites still reaches the centimeter level, which meets the application requirements of most missions. In general, the relative navigation accuracy of the deputy satellites is comparable with that of the chief satellites, regardless of scenario.
- Taking the scenario with a space-circle radius of 1 km as an example, the multi-satellite relative navigation accuracy is summarized in Figure 18. The multi-satellite relative navigation accuracy is significantly affected by the angle measurement accuracy. When the inter-satellite angle measurement accuracy is 0.1 deg, the multi-satellite relative navigation accuracy is only 1 m. However, when the inter-satellite angle measurement accuracy improves to 1 arcsec, the multi-satellite relative navigation accuracy is significantly affected by the range measurement accuracy. The multi-satellite relative navigation accuracy is better than 1 cm with a range measurement accuracy of better than 1 mm, and is maintained within 20 cm with a range measurement accuracy of 10 cm. Therefore, improving inter-satellite RF angle measurement accuracy is critical to further improving multi-satellite relative navigation accuracy.

## 5. Conclusions

We have proposed an innovative multi-satellite relative navigation scheme based on inter-satellite RF measurements for large-scale microsatellite formations. This scheme uses inter-satellite RF range and angle measurements. Only three chief satellites are required in this scheme, which significantly reduces the implementation difficulty of multi-satellite angle measurements. Simultaneously, based on the high-precision distributed multi-satellite RF range measurement scheme, a multi-satellite relative navigation algorithm has been developed and integrated with the measurement scheme. Numerical simulation results demonstrate the effects of the inter-satellite distance, GDOP value, range measurement accuracy, and angle measurement accuracy on the multi-satellite relative navigation accuracy. With the typical inter-satellite RF range and angle measurement accuracy, and an inter-satellite distance of around 1 km, the multi-satellite relative navigation accuracy reaches a level of ~30 cm, and the accuracy is comparable between the deputy satellites (which use range measurements) and the chief satellites (which use both range and angle measurements). Further, the multi-satellite relative navigation accuracy is robust to the number of chief satellites, demonstrating the incredible scalability of the proposed scheme. Finally, relative navigation accuracy can be improved to the centimeter level when more accurate angle measurement is provided using laser or infrared technology.

## Figures and Tables

**Table 1:** Advantages and limitations of different relative navigation approaches [1]. Reproduced with permission from IFAC Proceedings Volumes; published by Elsevier, 2011.

	Cost	Size, Weight, & Power	Range	Reliability	Accuracy
Radar	++	++	++	-	--
Laser	--	--	+	o	++
Binocular	+	o	-	-	+
3D-TOF camera	o	-	-	+	+
GNSS	++	+	++	+	+
RF ranging	++	+	+	+	+

++, very good; +, good; o, average; -, bad; --, very bad.

**Table 2:** Orbit elements of the seven-satellite space-circle formation (space-circle radius of 1 km).

Orbit Elements	a/km	e	*i*/deg	Ω/deg	*ω*/deg	*M*/deg
$S_{1}$	6878.14	0	97	0	0	0.8348
$S_{2}$	6878.14	7.2695 × 10^−5^	97	0.0073	180.0009	180.8348
$S_{3}$	6878.14	7.2694 × 10^−5^	97.0062	359.9964	60.0002	300.8341
$S_{4}$	6878.14	7.2694 × 10^−5^	96.9938	359.9964	299.9989	60.8355
$S_{5}$	6878.14	7.2695 × 10^−5^	97	359.9927	179.9991	180.8348
$S_{6}$	6878.14	7.2694 × 10^−5^	96.9938	0.0036	60.0011	300.8341
$S_{7}$	6878.14	7.2694 × 10^−5^	97.0062	0.0036	299.9998	60.8355

**Table 3:** Orbit elements of the seven-satellite space-circle formation (space-circle radius of 10 km).

Orbit Elements	a/km	e	*i*/deg	Ω/deg	*ω*/deg	*M*/deg
$S_{1}$	6878.14	0	97	0	0	0.8348
$S_{2}$	6878.14	7.2694 × 10^−4^	97	0.0727	180.0089	180.8348
$S_{3}$	6878.14	7.2694 × 10^−4^	97.0625	359.9637	59.9956	300.8347
$S_{4}$	6878.14	7.2694 × 10^−4^	96.9375	359.9637	299.9955	60.8349
$S_{5}$	6878.14	7.2694 × 10^−4^	97	359.9273	179.9911	180.8348
$S_{6}$	6878.14	7.2694 × 10^−4^	96.9375	0.0363	60.0045	300.8347
$S_{7}$	6878.14	7.2694 × 10^−4^	97.0625	0.0363	300.0044	60.8349

**Table 4:** Multi-satellite relative navigation results (simulated scenario: Cases a–d, space-circle formation radius of 1 km, $\sigma_{\varphi }=\sigma_{\theta }=0.01\;\deg,\sigma_{\rho }=1\;\mathrm{cm}$).

Scenario	$S_{1}/m$	$S_{2}/m$	$S_{3}/m$	$S_{4}/m$	$S_{5}/m$	$S_{6}/m$
Case a	0.144	0.341	0.349	0.226	0.363	0.372
Case b	0.141	0.215	0.345	0.230	0.366	0.366
Case c	0.120	0.243	0.214	0.228	0.369	0.368
Case d	0.128	0.226	0.206	0.235	0.368	0.366

**Table 5:** Simulation scenarios and simulation parameters.

Scenario	Simulation Parameters
$\alpha_{1}$	$\sigma_{\varphi }=\sigma_{\theta }=1\;\mathrm{arcsec},\sigma_{\rho }=0.01\;\mathrm{cm}$
$\alpha_{2}$	$\sigma_{\varphi }=\sigma_{\theta }=1\;\mathrm{arcsec},\sigma_{\rho }=0.1\;\mathrm{cm}$
$\alpha_{3}$	$\sigma_{\varphi }=\sigma_{\theta }=1\;\mathrm{arcsec},\sigma_{\rho }=1\;\mathrm{cm}$
$\alpha_{4}$	$\sigma_{\varphi }=\sigma_{\theta }=1\;\mathrm{arcsec},\sigma_{\rho }=10\;\mathrm{cm}$
$\beta_{1}$	$\sigma_{\varphi }=\sigma_{\theta }=0.01\;\deg,\sigma_{\rho }=0.01\;\mathrm{cm}$
$\beta_{2}$	$\sigma_{\varphi }=\sigma_{\theta }=0.01\;\deg,\sigma_{\rho }=0.1\;\mathrm{cm}$
$\beta_{3}$	$\sigma_{\varphi }=\sigma_{\theta }=0.01\;\deg,\sigma_{\rho }=1\;\mathrm{cm}$
$\beta_{4}$	$\sigma_{\varphi }=\sigma_{\theta }=0.01\;\deg,\sigma_{\rho }=10\;\mathrm{cm}$
$\gamma_{1}$	$\sigma_{\varphi }=\sigma_{\theta }=0.1\;\deg,\sigma_{\rho }=0.01\;\mathrm{cm}$
$\gamma_{2}$	$\sigma_{\varphi }=\sigma_{\theta }=0.1\;\deg,\sigma_{\rho }=0.1\;\mathrm{cm}$
$\gamma_{3}$	$\sigma_{\varphi }=\sigma_{\theta }=0.1\;\deg,\sigma_{\rho }=1\;\mathrm{cm}$
$\gamma_{4}$	$\sigma_{\varphi }=\sigma_{\theta }=0.1\;\deg,\sigma_{\rho }=10\;\mathrm{cm}$

## Data Availability

The data presented in this study are available on request from the corresponding author.
